# Congenital Obstructive Müllerian Anomaly: The Pitfalls of a Magnetic Resonance Imaging-Based Diagnosis and the Importance of Intraoperative Biopsy

**DOI:** 10.3390/jcm10112414

**Published:** 2021-05-29

**Authors:** Do Young Kim, Gina Nam, Sa Ra Lee, Sung Hoon Kim, Hee Dong Chae, Byung Moon Kang

**Affiliations:** 1 Department of Obstetrics and Gynecology, Asan Medical Center, University of Ulsan College of Medicine, 88, Olympic-ro 43-gil, Songpa-gu, Seoul 05505, Korea; kdy01179@naver.com (D.Y.K.); kimsung@amc.seoul.kr (S.H.K.); hdchae@amc.seoul.kr (H.D.C.); bmkang@amc.seoul.kr (B.M.K.); 2 Department of Obstetrics and Gynecology, Chung-Ang University Hospital, Chung-Ang University College of Medicine, Seoul 05505, Korea; ginanam@caumc.or.kr

**Keywords:** congenital obstructive anomaly, magnetic resonance imaging, biopsy, cervical dysgenesis

## Abstract

A retrospective cohort study of the concordance between the magnetic resonance imaging (MRI) diagnosis and final diagnosis in patients with Müllerian duct anomalies (MDAs) was conducted, and diagnostic clues were suggested. A total of 463 cases of young women who underwent pelvic MRIs from January 1995 to February 2019 at Seoul Asan Medical Center were reviewed. Interventions consisted of clinical examinations, abdominal or transvaginal/rectal ultrasound, MRI, and operative procedures, including hysteroscopy and laparoscopy. The concordance of the diagnosis between the results obtained with MRI and those obtained with surgeries was evaluated. It was found that a total of 225 cases (48.6%) showed genital tract anomalies on MRI. Among them, 105 cases (46.7%) underwent reconstructive surgery. Nineteen cases (8.4%) revealed discrepancies between the final diagnosis after surgery and the initial MRI findings and eleven cases (57.9%) had cervical anomalies. Incorrect findings associated with the MRIs were particularly evident in biopsied cases of cervical dysgenesis. A combination of physical examination, ultrasound, and MRI is suitable for preoperative work-up in the diagnoses of congenital obstructive anomalies. However, it is recommended that a pathologic confirmation of tissue at the caudal leading edge be made in obstructive genital anomalies, in cases of presumptive vaginal or cervical dysgenesis.

## 1. Introduction

Congenital Müllerian duct anomalies (MDAs) are relatively common, and diverse types of this condition have been reported [1]. MDAs are observed in 2–3% of fertile women, 3% of infertile women, and 5–10% of patients with repeated miscarriages [2], and various classifications have been proposed [3,4]. MDAs can also be categorized as obstructive and nonobstructive anomalies. Obstructive reproductive tract anomalies include an imperforate hymen, distal vaginal atresia or agenesis, transverse vaginal septum, cervical dysgenesis or agenesis, and an obstructive hemivagina with an ipsilateral renal anomaly (OHVIRA) [5], although an imperforate hymen is derived from the urogenital sinus and does not have a Müllerian duct origin.

Among various obstructive reproductive tract anomalies, differential diagnoses between cervical dysgenesis or agenesis and vaginal agenesis are especially important because the impact of these conditions on women’s sexual and reproductive potential can be significantly different. However, differentiation between the two is often difficult, even for experienced gynecologists and radiologists. The incidence of vaginal agenesis ranges from 1/4000 to 1/5000 [6,7]. For cervical agenesis or dysgenesis, the exact incidence is unknown, but it is considered to be approximately between 1/80,000 and 1/100,000 [6]. Cervical dysgenesis or agenesis is difficult to diagnose, and this can also contribute to its perceived rarity, with only approximately 200 cases reported to date. Moreover, 50% of affected patients have congenital vaginal agenesis [8]. 

In terms of treatment and prognosis of these two obstructive MDAs, treatment for vaginal agenesis is reconstructive vaginoplasty, after which, patients can plan a pregnancy [9]. However, the classical treatment of congenital cervical agenesis or dysgenesis has been total hysterectomy due to the increased risk of infection, sepsis, and even death after canalization surgery [10,11,12,13,14]. Nonetheless, many cases of successful reconstructive surgery, even with vaginal [15] or minimally invasive laparoscopy [16,17], and some instances of successful pregnancies and deliveries after surgery in patients with cervical agenesis or dysgenesis, have also been reported [18]. Therefore, conservative surgical management, such as uterovaginal or uterovestibular anastomosis, has been considered as a treatment option by several authors [18,19]. However, consensus on the optimal treatment for these rare obstructive MDAs remains controversial.

An accurate diagnosis of the type of MDA is important to determine the appropriate treatment option, timing or type of surgery, and counseling about reproductive and sexual outcomes. Symptoms, physical examination, imaging modalities, including ultrasonography (US), magnetic resonance imaging (MRI), hysterosalpingography, and diagnostic operative procedures including hysteroscopy and laparoscopy, all help in the differential diagnosis of MDAs. In terms of imaging studies, US may be the first screening tool, and the introduction of three-dimensional US has increased the accuracy comparable to that of pelvic MRI; however, it is not very reliable in the diagnosis of cervical atresia or dysgenesis [20]. The gold standard imaging modality for MDAs is known to be pelvic MRI. MRI is a well-established, excellent diagnostic tool in the evaluation of the female pelvis and has also been a preferred imaging modality for pediatric patients when transvaginal or transrectal US is not available. Compared with US or computed tomography (CT), MRI provides greater tissue detail and focuses more on the contrast of soft tissue; therefore, MRI has been considered to be superior to US or CT for examining the cervicovaginal anatomy or lesions [21,22]. 

Interpretation of an MRI finding of a cervical anomaly is based on the contour and signal intensity of the genital tract in multiplanar images [21]. However, some discrepancies between MRI findings and surgical diagnoses have been documented, especially in cervicovaginal anomalies [23,24,25].

This study was, therefore, performed to evaluate the accuracy of MRI-based diagnoses for MDAs and to reinforce the importance of intraoperative biopsies for the exact diagnosis and selection of the proper surgical methods for obstructive MDAs. In this study, we performed the diagnosis and classification of MDA as per the guidelines of the American Society for Reproductive Medicine (ASRM), which remains the standard till date [26]. Based on our experience, we were able to reduce re-obstructions by applying appropriate reconstructive surgery according to an accurate diagnosis based on intraoperative biopsy results relating to whether the obstructive MDA was vaginal or cervical dysgenesis or agenesis.

## 2. Materials and Methods

A total of 463 cases of young women, aged 9–25 years, who underwent pelvic MRIs for various reasons from January 1995 to February 2019 at Seoul Asan Medical Center, were retrospectively reviewed. The indications for MRI were the clarification of imaging findings of suspected MDAs on US or CT in cases of clinical symptoms, such as amenorrhea, pelvic pain, dysmenorrhea, and dyspareunia. Patients were excluded from the study if they were not diagnosed with genital tract anomalies as determined by MRI. Among 463 patients, a total of 225 patients diagnosed with genital tract anomalies, as indicated by the final analysis using MRI, were included in the study. The final diagnosis of the MDAs, the postoperative diagnosis after hysteroscopic or pelviscopic reconstructive surgery, and the preoperative MRI findings in patients who underwent reconstructive surgery were compared. Patients with classical Mayer–Rokitansky–Küster–Hauser (MRKH) syndrome, with or without accompanying anomalies, were excluded. In this study, we specifically focused on the preoperative description of MR images that showed vaginal or cervical dysgenesis or agenesis. We compared the final surgical diagnosis of the obstructive MDAs with the preoperative MRI diagnosis to determine the accuracy of MRI in the diagnosis of obstructive MDAs. 

### 2.1. MRI Assessment

All patients underwent MRI prior to surgery using 1.5T (Achieva; Siemens Healthcare, Erlangen, Germany) or 3.0T (Skyra; Siemens Healthcare or Ingenia, Philips Healthcare, Best, Netherlands) systems with a phased-array body coil. Sequence selection included axial, coronal, and sagittal T2-weighted images, T1-weighted images, diffusion-weighted images of the pelvis, and T2-weighted turbo spin echo images covering the abdomen and pelvis. Intravenous gadolinium administrations inducing T1-weighted, contrast images were also obtained. All MR images were preoperatively assessed by experienced radiologists at our hospital who were blinded to the surgical findings and final diagnosis. The features of the genital tract, including the congenital anomaly of the uterus, cervix, vagina, and ovaries, were also interpreted. The contour, shape, and uterine wall thickness, as well as the signal intensity of the hematometra or hematocolpos were described. The preoperative MRI diagnosis was compared with the final diagnosis after surgery.

### 2.2. Surgical Assessment 

All patients were examined at the outpatient clinic before surgical assessment. The timing and method of surgical procedures were determined on the basis of the presumptive diagnosis, combined with the clinical diagnosis and imaging study results. All surgeries were performed by a single gynecologist (B.M.K.) with experience of more than 30 years in adolescent gynecology, in our adolescent gynecology clinic, designated as a training center for the surgery of congenital MDAs by the International Federation of Pediatric and Adolescent Gynecology (FIGIJ) in 2018. Surgeries performed on each case of the obstructive reproductive tract anomalies were as follows. Cruciate and ovoid incisions to the thin imperforate hymen were made to open the vaginal orifice. Continuous locking sutures with 2-0 Vicryl (Ethicon Inc., Somerville, NJ, USA) were used for hemostasis of the edge of the circumferentially incised hymen. The thin transverse vaginal septum could be directly resected followed by end-to-end anastomosis of the lower and upper vagina [27]. If the normal vaginal tissue with the thick septum was not enough, the septum was divided into a distal section in an “X” fashion and a proximal section in a “+” fashion [5]. Created leaflets were interdigitated to bridge a larger distance [5]. 

In cases of vaginal agenesis, the vagina was first gradually dilated with the Frank method, which was successful in creating a functional vagina in most cases, as reported by other researchers [28,29]. However, when the Frank method fails, surgical interventions, including the McIndoe technique [30] or Davidov surgery [31], were performed. In undistinguishable cases of vaginal or cervical dysgenesis, we chose the surgical approach. First, we obtained access to the lesion with the hematoma under US guidance; as soon as we noticed the old blood spilling from the lesion on aspiration, we performed an intraoperative tissue biopsy at the caudal leading edge of the hematoma mass for confirmation of the presence of vaginal or cervical tissue in six cases of obstructive MDAs. After confirming whether the tissue was from the cervix or vagina, we proceeded with the reconstructive surgery accordingly. In cases of cervical agenesis or dysgenesis, the atretic portion of the cervix was opened until the uterine cavity was encountered. The surgical technique required a complete dissection of the rectouterine and vesicouterine space to expose the vagina, allowing circumferential anastomosis of the vagina to the lower uterine segment. 

In terms of the extent of tissue dissection, vaginal dissection with the Davidov method, a vaginoplasty for vaginal agenesis, was performed in the usual manner. However, in cases of cervical dysgenesis or agenesis, the vaginal lateral dissection was extended deeper, in a more lateral direction compared to that in vaginal agenesis, to decrease re-obstruction and avoid re-operation. 

This study was approved by the Asan Medical Center Institutional Review Board (approval No. 2020-1035). Informed consents were waived by our Institutional Review Board because of the retrospective chart-review study design.

### 2.3. Statistical Analysis 

All data are presented as frequencies and percentages. A chi-square test was performed to compare the proportions of the categorical variables between the two groups. A *p*-value < 0.05 was considered to be statistically significant. Statistical analyses were performed using R, a language and software environment for statistical computation (R Foundation for Statistical Computing, Vienna, Austria) [32].

## 3. Results

The mean age of the initial cohort (n = 463) was 18.05 ± 4.59 years. Among the 463 cases, 225 (48.6%) were interpreted as having genital tract anomalies on MRI. The mean age of the included patients (n = 225) was 17.30 ± 4.21 years. In terms of diagnosis, the MRKH syndrome was the most common anomaly (75 cases, 33.3%). Of the 75 cases, 42 (33.3%) had uterine didelphys, including OHVIRA, and 27 (12.0%) exhibited anomalies of the hymen and lower one-third of the vagina, including an imperforate hymen, distal vaginal atresia or agenesis, and a transverse vaginal septum (Table 1). Among the 225 cases, 105 (46.7%) underwent reconstructive surgery according to their diagnosis. In cases of MRKH, the Frank method was performed in 52 cases (69.3%) instead of reconstructive surgery.

**Figure 1:**
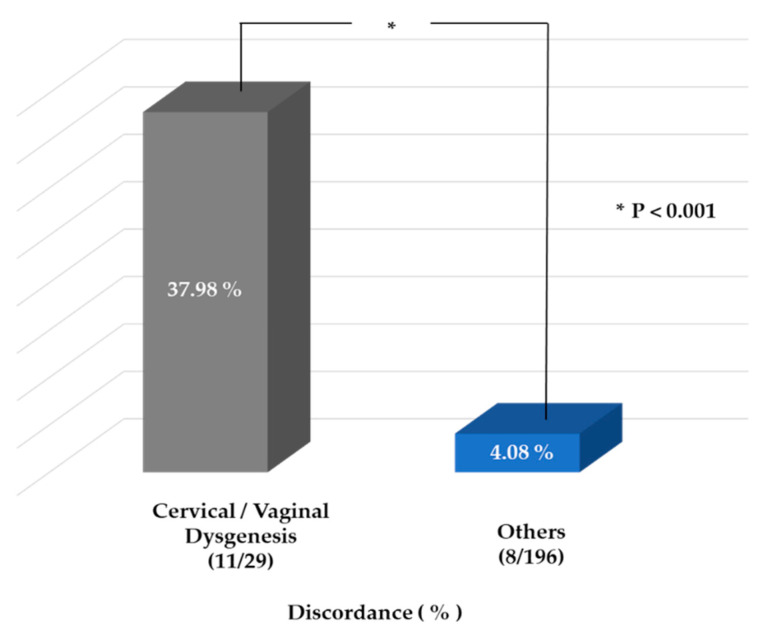
A graph of the discordance between the magnetic resonance imaging-based diagnosis and the final diagnosis in patients with genital tract anomalies (* *p* < 0.001, chi-square test).

Table 2 shows each case of disagreement between the preoperative MRI findings and the final surgical diagnosis. We defined final surgical diagnoses on the basis of pathologic confirmation by intraoperative tissue biopsies at the caudal leading edge of the hematoma lesions and findings of diagnostic hysteroscopy and pelviscopy. Nineteen of the 225 cases (8.4%) showed a discrepancy between the MR interpretation and the final diagnosis. Among the considered patients, the outcomes of the MRI-based diagnosis did not match the final diagnosis in patients with cervical anomalies in cases 1, 8, 9, 11, and 13–19. The discordance rate for cervical or vaginal dysgenesis was significantly higher (57.9% (11 out of a total of 19 cases)) than that of other types of MDAs (4.08% (8 of 196 cases), *p* = 8.36 × 10^−9^) (Table 2, Figure 1).

**Figure 2:**
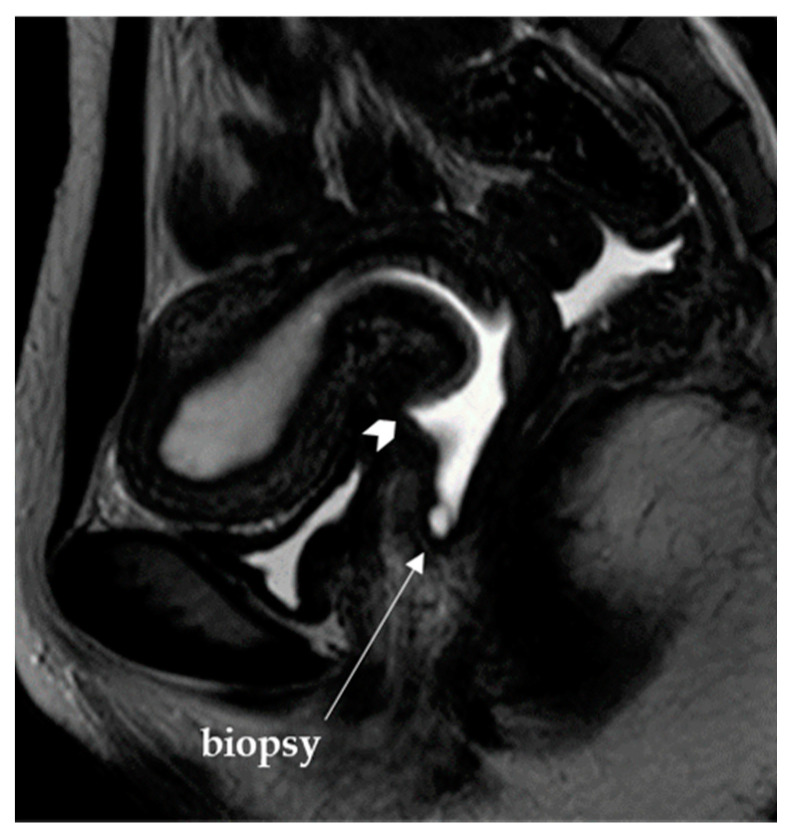
The sagittal T2-weighted magnetic resonance image shows a definite contour of the cervix (arrowhead), suggesting vaginal agenesis with fluid collection in Patient 15 (Table 2). However, the intraoperative biopsy, which was performed at the caudal leading edge (arrow), revealed the presence of cervical gland and stroma material.

For example, in Patient 15, preoperative MR images showed a clear cervical contour and were assumed to represent vaginal stenosis with a presumed hematocolpos (hematoma in vagina); therefore, a vaginoplasty for vaginal agenesis was planned (Figure 2). However, an intraoperative tissue biopsy at the caudal edge of the hematoma lesion, producing presumptive vaginal tissue, revealed the presence of cervical gland and stroma material. Therefore, the final diagnosis of the case was changed to cervical dysgenesis accompanied with vaginal agenesis. In addition, diagnostic hysteroscopy showed no resistance when the telescope entered the uterus-like lesion, in which this surgical finding also supported the final diagnosis, cervical dysgenesis accompanied with vaginal agenesis. 

## 4. Discussion

Our study showed that the concordance rate between the clinical presumptive diagnoses and MRI interpretation was 81.9% (86 out of 105) for patients who underwent reconstructive surgery, and the discordance rate was 18.1% (19 out of 105). Eleven cases were misdiagnosed as cervical atresia in 29 patients with cervicovaginal atresia.

Clinical examination, US, MRI, hysteroscopy, and laparoscopy can help diagnose MDAs. There have been many studies evaluating the accuracy of MRI and US in determining the anomalies of the female genital tract. Earlier studies described MRI as a highly accurate tool for the evaluation of MDAs. Pellerito [33] reported an accuracy of 100% for MRI and 92% for US for the evaluation of MDAs in 12 cases. Pompili et al. [34] found a sensitivity and specificity of 100% using MRI for diagnosing MRKH in 56 patients. Preoperative MRI also produces excellent manifestations and accurate diagnoses in terms of the classification of oblique vaginal septum syndrome [35]. MRI was superior to US in diagnosing uterovaginal malformations in a subgroup of women with MRKH (n = 7) [36]. However, in another report, Soares et al. [37] described a sensitivity of 44.4% and a specificity of 100% for diagnosing uterine malformations with US. Lermann et al. [38] reported that a combination of clinical examination and US is as accurate as MRI alone. A high correlation between the diagnosis by MRI and surgical findings was demonstrated in previous studies. MRI results concurred with the diagnosis of 24 out of 24 cases of surgically proved anomalies and demonstrated a sensitivity and specificity of 100% in the diagnosis of a septate uterus [33]. Santos et al. [23] found that MRI was consistent with surgical findings in 88.5% of cases.

However, there has been a study revealing that the accuracy of MRI differs between uterine and vaginal anomalies [24]. An excellent agreement between MRI and clinical diagnoses in uterine anomalies has been noted; however, 2 out of 11 incorrect MRI diagnoses of vaginal anomalies were found in two menstruating, adolescent patients with cerebral palsy. In both patients, the diagnosis of vaginal stenosis was inferred from the presence of a vaginal cavity distended with urine due to spasticity of the pelvic floor. There was uterine didelphys with an obstructed left hemivagina case on the pelvic MRI, but the surgical finding was an obstructed, non-communicating left rudimentary horn [23]. Assessment of the cervix and vagina on MRI is not usually requested because they can be examined clinically through direct visualization and misdiagnosed on MRI, as has been indicated in these reports.

Thus far, no study has reported on the correlation between the MRI and final diagnoses using an intraoperative tissue biopsy for the confirmation of cervical or vaginal tissues in cases of presumptive cervicovaginal dysgenesis. The emphasis of this study is that reconstructive surgery should be planned according to the pathologic confirmation of the tissue. Because there is a paucity of accuracy when diagnosing cervicovaginal anomalies in MRI, a precise diagnosis is required through the histological confirmation of the caudal leading edge during surgery. Based on the MR image, the biopsy should be performed at the leading edge of the hematometra or hematocolpos lesion, and according to the result of the pathology, the final diagnosis should be confirmed. Kimble et al. [39] also stated the limitations of MRI and described the histological specimen results in their two cases of partial cervical agenesis and complete vaginal atresia. The presence of the endocervix, but absence of the ectocervix, was shown in the histological specimen after hysterectomy [39]. 

Prior to discussion about surgical treatment options, we should be better informed regarding the differences in the tissue compositions of the cervix and vagina. Vaginal tissue is much more distensible, and therefore, a larger amount of hematoma can be retained. In contrast, the hard tissue nature of the cervix enables only a small amount of hematoma to be retained in the endocervical canal. This can lead to an earlier diagnosis of cervical or vaginal dysgenesis as there are earlier and more severe presentations of symptoms, such as cyclic abdominal pain, associated with even a small amount of hematoma, compared with a transverse vaginal septum or imperforate hymen, both of which manifest delayed symptoms. The tissue compositions of the uterine body and cervix are also different in that the uterine body is mostly composed of muscular layers, in contrast to the cervix, which is composed of collagen and elastin, and in which the muscle components are below 10%. In addition, some degree of hardness and resistance can be encountered in the narrow endocervical canal when the telescope enters into this canal during hysteroscopy. This may also explain why canalization techniques are less invasive [40]; however, they are linked to a high risk of restenosis of the cervix, up to 40–60% [6]. Hence, uterovaginal or uterovestibular anastomosis should take preference over canalization techniques for cervical agenesis or dysgenesis [15,18].

**Figure 3:**
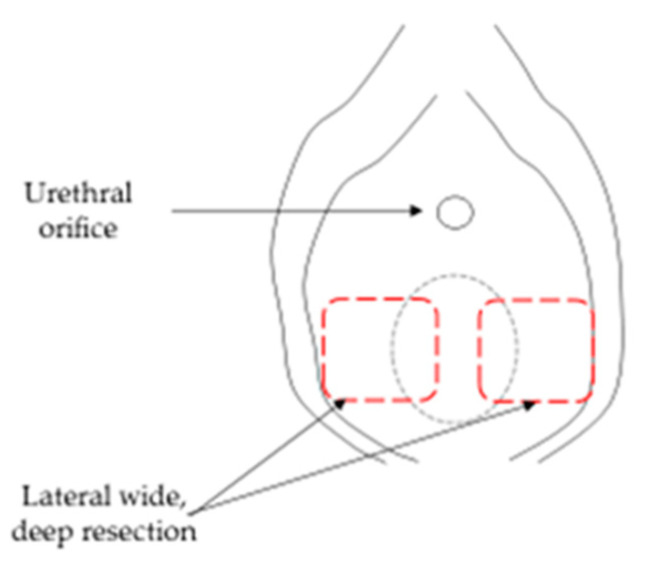
Lateral wide, deep resection for operating vaginal agenesis.

In terms of treatment options for vaginal agenesis, nonsurgical vaginal canalization is the first-line approach. Frank has described a conservative method of vaginal dilatation using Pyrex tubes [41]. Several surgical methods of vaginoplasty have also been developed. McIndoe used a skin graft with a mold to canalize the vagina, but it has been found that restenosis was a common complication [42,43]. In our experience, there have been no cases of postoperative re-obstruction in patients with vaginal agenesis who underwent lateral deep resection. The vaginal dissection was initially performed to enter the plane between the bladder anteriorly and the rectum posteriorly. The lateral wide, deep resection prevents re-obstruction after surgery (Figure 3). 

Deffarges et al. [18] confirmed that cervical atresia was successfully treated by uterovaginal anastomosis even when associated with vaginal agenesis. An inverted U-shaped incision was made on the perineum or vaginal tissue for anastomosis with a pulled-down uterus [18]. Laparoscopically assisted uterovestibular anastomosis was also performed [44]. By preserving the uterus, this process allows the patient to become pregnant and have a successful pregnancy outcome. The uterovaginal anastomosis was performed in our patients with tissue-confirmed cervical agenesis or dysgenesis. Thus, the effective conservative treatment and proper surgical option can be achieved by accurate preoperative and intraoperative diagnoses. We also support conservative uterovestibular or uterovaginal anastomosis after confirmation of the presence of cervical agenesis with an intraoperative tissue biopsy, as there were no cases of infection, sepsis, or death in our institution [15]. To prevent re-stenosis after the surgery and preserve future fertility, we recommend more lateral deep dissection before anastomosis for cervical dysgenesis. 

This study has some strengths. It considers a relatively large number of cases of congenital uterovaginal anomalies through diagnosis to treatment. However, there are several factors that contribute to the discordance between MRI readings and surgical outcomes. This could be considered as a major limitation of the study. First, since cases of cervical dysgenesis are rare among MDAs, there are only few references for radiologists to carry out further analysis. Second, three-dimensional sequences are considered as the most effective approach for the diagnosis of MDAs; however, MRI is not a three-dimensional imaging technique. Third, interpretational errors can be caused due to poor depictions of some structures on the MRI. These errors could be attributed to small size (e.g., atretic uterus), physiological status (e.g., collapsed vagina) because of a large amount of hematoma persistent for a long period of time, or deformations from previous surgeries (e.g., stenosis or adhesion formation). These factors could result in suboptimal signals, resolution, or visualizations on the MRI. Lastly, there could be an interobserver bias owing to the interpretation and experience of each radiologist. MRI is usually interpreted by two radiologists from the radiology department. One of the two radiologists takes the initial reading and then the senior radiologist, who specializes in pelvic radiography, reviews and provides a final diagnosis. The images from the radiology department are diagnosed in a pragmatic manner in most hospitals, and therefore attain excellent interobserver variability.

## 5. Conclusions

MRI is an excellent imaging modality for the diagnosis of MDAs. However, clinicians should be aware of this modality’s limitations, especially in cases where cervical or vaginal dysgenesis is suspected. Intraoperative pathologic confirmation of the tissue through a biopsy of the caudal leading edge of the hematoma is often essential for the accurate diagnosis and proper surgical treatment of a congenital obstructive genital anomaly, especially in cases of cervical dysgenesis and vaginal dysgenesis.

## Figures and Tables

**Table 1:** The diagnosis of pelvic magnetic resonance imaging in 225 patients with genital tract anomalies.

Diagnosis	Cases (%)
MRKH (with cervical dysgenesis)	75 (33.3)
Anomalies of the hymen and lower one-third of the vagina Imperforate hymen Distal vaginal atresia Transverse vaginal septum Imperforate hymen or transverse vaginal septum *	15 (6.7) 10 (4.4) 2 (0.9) 2
Hypoplastic uterus (POI, Kallmann syndrome)	9 (4.0)
Arcuate uterus	5 (2.2)
Septate uterus	4 (1.8)
Unicornuate uterus	8 (3.6)
Bicornuate uterus	13 (5.8)
Uterine didelphys (include OHVIRA)	42 (18.7)
DSD (AIS, Swyer syndrome)	26 (11.6)
Mixed gonadal dysgenesis	4 (1.8)
Gonadal dysgenesis	4 (1.8)
5α-reductase deficiency	3 (1.3)
Others †	5 (2.2)
Total	225 (100)

MRKH, Mayer–Rokitansky–Küster–Hauser; POI, premature ovarian insufficiency; OHVIRA, obstructed hemivagina with ipsilateral renal anomaly; DSD, disorders of sexual development; AIS, androgen insensitivity syndrome; *, both imperforate hymen and transverse vaginal septum were reported in magnetic resonance imaging; †, three cases of variants of Turner syndrome, one case of cervical cyst, and one case of vesicocervical fistula.

**Table 2:** Comparisons between the MRI and final diagnoses in 19 patients with discrepancy results.

Patient No.	MRI Diagnosis	Age	Final Diagnosis
1	Imperforate hymen	12y	Cervical anomaly with a vaginal septum
2	Septate vagina	15y	Cervical cyst
3	Uterine and gonadal aplasia	17y	Premature ovarian failure
4	Degenerative myoma in the uterus	14y	Uterine didelphys with non-communicating horn
5	Hysterectomy state	14y	MRKH
6	True hermaphroditism	16y	MRKH
7	MRKH	15y	46, XY DSD male pseudohermaphroditism
8	Vaginal stenosis	16y	Cervical agenesis with transverse vaginal septum
9	Lower vaginal agenesis	12y	Cervical dysgenesis
10	MRKH	15y	46, XY DSD male pseudohermaphroditism
11	Imperforate hymen	22y	Cervicovaginal agenesis
12	True hermaphroditism	17y	MRKH (Bx:No testicular tissue)
13	Hypoplastic or aplastic vagina with hematocolpometra	13y	Cervical dysgenesis
14	Stricture of the lower vagina	13y	Cervical dysgenesis
15	Vaginal stenosis	13y	Cervical dysgenesis with vaginal agenesis
16	Bicornuate uterus with an imperforate hymen	14y	Bicornuate uterus with cervical anomaly
17	Uterine didelphys with a left hemi-vaginal septum	15y	Uterine didelphys with left cervical agenesis
18	Vaginal atresia	12y	Vaginal atresia with cervical agenesis
19	Obstructive hemivagina with ipsilateral renal anomaly	12y	Uterine didelphys with unilateral cervical dysgenesis, vaginal agenesis, renal agenesis

MRKH, Mayer–Rokitansky–Küster–Hauser syndrome; DSD, disorders of sexual development.

## Data Availability

The excel data used to support the findings of this study were supplied by Sa Ra Lee under license, and requests for access to these data should be made to S.R.L. leesr@amc.seoul.kr.
